# Understanding and mitigating thiaminase activity in silver carp

**DOI:** 10.1016/j.crfs.2023.100502

**Published:** 2023-04-07

**Authors:** Patricia C. Wolfe, Amber M. Tuske, Donald E. Tillitt, Fred Allen, Katie A. Edwards

**Affiliations:** a Department of Pharmaceutical Sciences, Binghamton University, Binghamton, NY, 13902, USA; b U.S. Geological Survey, Columbia Environmental Research Center, Columbia, MO, 65201, USA; c RADii Solutions, LLC, Princeton, NJ, 08540, USA; d Carpe Eat'm, LLC, Paducah, KY, 42001, USA

**Keywords:** Thiaminase, Invasive species, Thiamine deficiency, Food processing, Silver carp

## Abstract

A deficiency of thiamine (vitamin B1), an essential cofactor for enzymes involved in metabolic processes, can be caused by the enzyme thiaminase. Thiaminase in food stocks has been linked to morbidity and mortality due to thiamine depletion in many ecologically and economically important species. Thiaminase activity has been detected in certain bacteria, plants, and fish species, including carp. The invasive silver carp (*Hypophthalmichthys molitrix)* presents an enormous burden to ecosystems throughout the Mississippi River watershed. Its large biomass and nutritional content offer an attractive possibility as a food source for humans, wild animals, or pets. Additionally, harvesting this fish could alleviate some of the effects of this species on waterways. However, the presence of thiaminase would detract from its value for dietary consumption. Here we confirm the presence of thiaminase in several tissues from silver carp, most notably the viscera, and systematically examine the effects of microwaving, baking, dehydrating, and freeze-drying on thiaminase activity. Certain temperatures and durations of baking and microwaving reduced thiaminase activity to undetectable levels. However, caution should be taken when carp tissue is concentrated by processes without sufficient heat treatment, such as freeze-drying or dehydration, which results in concentration, but not inactivation of the enzyme. The effects of such treatments on the ease of extracting proteins, including thiaminase, and the impact on data interpretation using the 4-nitrothiophenol (4-NTP) thiaminase assay were considered.

## Highlights

• Thiaminase I is an enzyme that breaks down the essential vitamin thiamine (vitamin B1).
• Thiaminase I activity was found in invasive silver carp (*Hypophthalmichthys molitrix*).
• We tested thiaminase I activity before and after various common food processing steps.
• Processes without sufficient heat treatment dehydrate and concentrate enzyme activity.
• Certain microwaving or baking times and temperatures effectively reduced the activity.

## Graphical abstract

**Figure:**
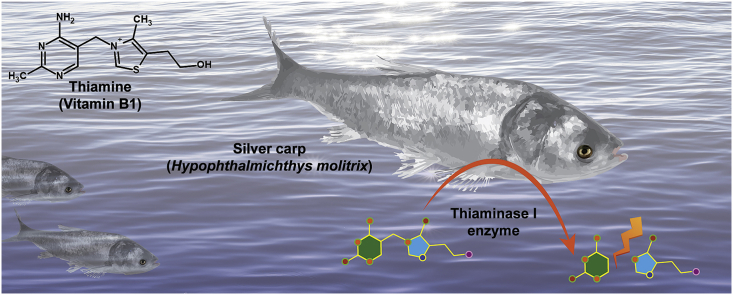

## 1 Introduction

The water-soluble vitamin thiamine (vitamin B1) is essential to all forms of life. Its active diphosphorylated version, thiamine pyrophosphate (TPP), is an essential cofactor in carbohydrate and amino acid metabolism; thus, thiamine deficiency leads to severe health consequences. Clinically relevant thiamine deficiency commonly manifests as neurological symptoms and results from a thiamine-deficient diet or defects in thiamine transport from the gut or into tissues (Irle and Markowitsch, 1982; Kritikos et al., 2017; Marks et al., 2011). Thiamine is unstable to various conditions found in food processing, including alkaline conditions, elevated temperatures, and the presence of sulfites (Coelho, 2002; Kritikos et al., 2017). This has contributed to thiamine losses in animal feed leading to thiamine deficiency. Low thiamine is a common source of food product recalls (Chang et al., 2017; Kritikos et al., 2017; Markovich et al., 2014; Marks et al., 2011; Singh et al., 2005). Thiamine deficiency can also be triggered by the presence of enzymes known as thiaminases that break down thiamine before it can be used (Harder et al., 2018; Hrubša et al., 2022; Kritikos et al., 2017; Neilands, 1947).

Two types of thiaminase have been described, both of which break down thiamine by catalyzing the exchange of a nucleophilic cosubstrate for the thiazole ring. Thiaminase II is found in many bacteria and uses water as its hydrophilic cosubstrate (Toms et al., 2005). Thiaminase I is found in certain bacteria and has also been described in some species of fish, plants, and at least one insect (Boggs et al., 2019; Kraft et al., 2014; McCleary and Chick, 1977; Nishimune et al., 2000; Richter et al., 2023). It catalyzes a similar reaction but uses various cosubstrates, such as pyridine, cysteine, pyridoxine, and nicotinic acid (Fujita, 1972; Fujita et al., 1952).

In the 1930s, several fox farms experienced a fatal paralytical disorder among their animals that began soon after substituting raw fish for a portion of their feed, which resolved when the fish was removed from their diets. Thiamine deficiency was determined to be the basis of the morbidity, but the basis of the deficiency was not a lack of dietary thiamine but rather a degradation of thiamine by a then-unknown factor (Green et al., 1941). Since then, early studies found that thiaminase in raw clams was sufficient to degrade more than eight times the minimum daily amount of thiamine in the human diet (Melnick et al., 1945). Similar incidents of disease due to thiaminase-linked thiamine deficiency have been noted in cats consuming raw carp or herring (Smith and Proutt, 1944), captive sea mammals consuming raw fish (White, 1970; White and Francis-Floyd, 1988), American alligators fed gizzard shad (*Dorosoma cepedianum*) (Ross et al., 2009), and ruminants or horses consuming bracken fern (*Pteridium aquilinum*) or horsetail (*Equisetum arvense*) (Klebesadel and Mitchell, 1964; Stegelmeier et al., 2013). Moreover, Cayuga Syndrome, which causes 98–100% mortality in the affected fry of land-locked salmon, has been linked to maternal consumption of thiaminase-containing invasive alewife (*Alosa pseudoharengus*) (Fisher et al., 1995). Thiamine deficiency in fish has been linked to the presence of thiaminase I enzymes (Honeyfield et al., 2002, 2005; Houde et al., 2015; Tillitt et al., 2005).

Silver carp (*Hypophthalmichthys molitrix*) is one of four invasive species of heavy-bodied freshwater cyprinid fishes in the United States, known collectively as ‘Asian carp’ or ‘Invasive carp’ (Ferguson et al., 2020; Li et al., 2021). The other three species are Bighead carp (*Hypophthalmichthys nobilis*), Black carp (*Mylopharyngodon piceus*), and Grass carp (*Ctenopharyngodon idella*). The fish originated in Asia and were introduced into freshwater aquaculture ponds in the midwestern United States approximately 50 years ago. *H. molitrix* have proliferated in the Mississippi River, its tributaries, and associated lakes. Consumption by humans and animals has been explored as a possible solution to alleviate the diverse ecological problems posed by this invasive fish (Li et al., 2021). In June 2022, the Illinois Department of Natural Resources renamed Asian carp to ‘Copi’, stemming from the word copius, in an effort to improve public perceptions and interest in it as a food source (Illinois Department of Natural Resources, 2022). Currently, several offerings of dog treats and foods are commercially available. The flesh of these invasive carp is considered highly nutritious as a wild protein source with an appreciable concentration of omega-3 fatty acids, collagen, vitamins, and minerals and lower heavy metal concentrations than saltwater fish (Acharya et al., 2022; Ferguson et al., 2020). However, several literature reports have noted the presence of thiaminase activity in *H. molitrix* and other carp species (Arsan and Malyarevskaya, 1969; Bos and Kozik, 2000; Gnaedinger and Krzeczkowski, 1966; Greig and Gnaedinger, 1971; Krampitz and Woolley, 1944; Lee, 1948; Masako et al., 1994; Sealock et al., 1943; Spitzer et al., 1941). We thus pursued clarity on the levels and location of thiaminase activity in *H. molitrix* and assessed processing methods to deactivate the thiaminase in these fish products.

Though thiaminase I across its various sources is consistent in its chemical action and requirement for a nucleophilic cosubstrate, the sensitivity of its activity to temperature varies depending on its source (Gnaedinger and Krzeczkowski, 1966; Hilker and Peter, 1966; Nishimune et al., 2000). For example, in an early study, thiaminase in raw carp, shad, and alewife was found to be inactivated after 5 min at 180 °F (Gnaedinger and Krzeczkowski, 1966), while the thiaminase in African silkworm pupae appears more resistant to heat, such that roasting the pupae and cooking them in stew failed to destroy the thiaminase and led to seasonal ataxia in humans consuming them (Nishimune et al., 2000). Therefore, it is essential to determine more clearly the conditions under which the possible activity of thiaminase in *H. molitrix* is reduced to safe levels. The primary aim of this study was to establish baseline levels of thiaminase for fresh and frozen raw ground silver carp meat and different body parts and then to determine the effect of heat treatment or cooking method on reducing the thiaminase level to ensure that the product is suitable for consumption by humans or animals.

## 2 Materials and methods

### 2.1 Materials

Potassium phosphate, sodium chloride, and 4-nitrothiophenol (4-NTP) 96% were purchased from Alfa Aesar; tris (2-carboxyethyl)phosphine hydrochloride (TCEP) was purchased from TCI America. Thiamine hydrochloride and dimethylsulfoxide (DMSO) were purchased from Sigma Aldrich. HALT™ protease inhibitor cocktail was purchased from ThermoScientific. All buffers were made using HPLC-grade water purchased from VWR.

The recombinant *Clostridium botulinum* thiaminase I was overexpressed in *Escherichia coli* and was purified using a Ni-chelate resin using previously reported procedures (Edwards et al., 2019; Sannino et al., 2018). The concentration of the purified enzyme was determined using the Micro BCA™ assay (Thermo Fisher), and purity was assessed using SDS-PAGE with Coomassie staining.

### 2.2 Fish source

**Fig. 1:**
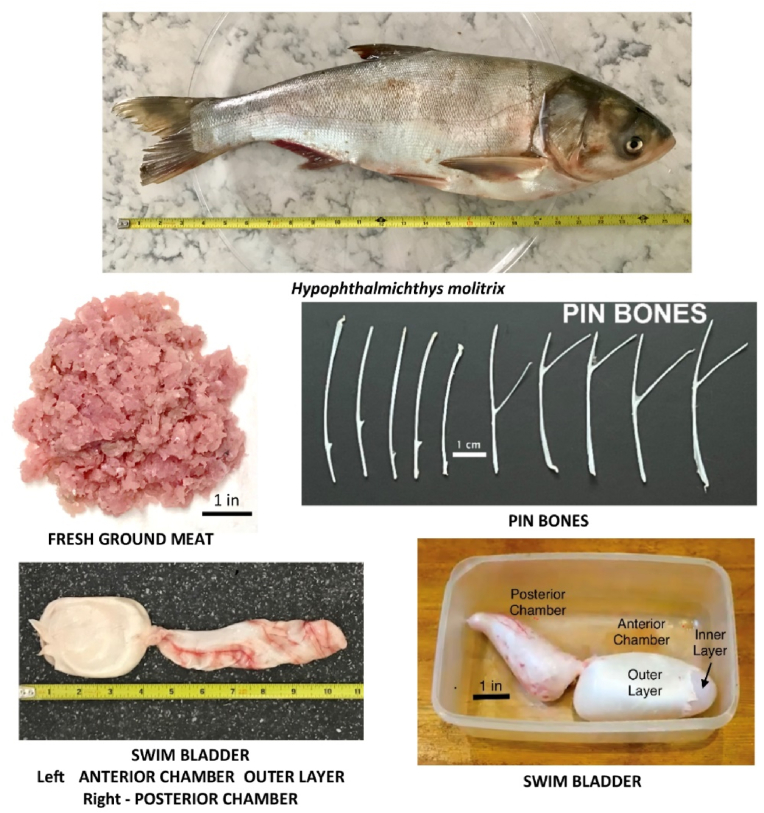
Images of an individual *Hypophthalmichthys molitrix* fish and select raw samples including fresh ground meat, pin bones, and swim bladder (with anterior and posterior chambers and inner and outer layers labeled). Each individual image has its own scale included within the image.

Silver carp were collected from Two Rivers Fisheries (TRF), Inc., in Wickliffe, KY (36.9700°N, 89.0637°W), located at the confluence of the Mississippi and Ohio Rivers and 60 miles west of the north end of Kentucky Lake and Lake Barkley. The fish were caught using gill nets by commercial fishers and brought to TRF from the local rivers and lakes for processing, including heading, gutting, grinding, and flash freezing. Freshly caught and flash-frozen silver carp were processed to extract various body parts (e.g., viscera, swim bladder, organs). The skin and bones were removed, and the flesh was ground to homogenize. Selected images of typical *H. molitrix* processed at TRF and representative samples are presented in Fig. 1. Fresh and frozen ground meat was randomly sampled from batches originally comprising several thousand pounds of silver carp. After harvesting, specimens from various tissues were used raw or frozen without further processing or treated by baking, microwaving, dehydration, or a combination of these methods at different temperatures and lengths of time. All processing that involved ground meat (‘flesh’) was done with 1–2 lb samples taken from 20 lb bags obtained from TRF. The skinless and boneless ground meat left after processing headed and gutted fish is referred to as ‘flesh’ within. This involves removing the skin and bones and converting muscle tissue into ground meat. Parts of fish – e.g., bones and skin – as well as stickwater were obtained from large scale grinding of ∼1000 × 10 lb fish, headed and gutted, yielding ∼3000 lb batches (60 × 20 lb bags) of ground meat. Stickwater is the liquid that drains from the raw or frozen ground fish upon thawing and is sometimes used in commercial animal meals. Bladders were removed from raw fish, and 50 pieces were separated into inner and outer portions processed for this study. The samples were shipped overnight on dry ice to the laboratory, where they were stored at −80 °C until thiaminase extraction and assay.

We tested baseline levels of thiaminase activity in fresh and frozen raw ground meat and different body parts to determine the effect of heat treatment or cooking method on reducing the thiaminase activity level. Temperature and time were considered for three processes: dehydration, baking, microwaving, and freeze-drying. These processing methods are commonly employed in industrial settings to manufacture dry pet foods or treats. Table S2 lists the samples analyzed in the study. The shipment to the laboratory included four replicate samples of each tissue and processing step, except for pin bones which were from a single fish. For all conditions except for bladders and pin bones, four samples, with each sample filling approximately the volume of a 15 mL centrifuge tube, were taken from large batches of fish, as described above. For bladders, inner and outer bladders were separated from raw fish as described above. For pin bones, a single tube of pin bones was supplied for this analysis.

### 2.3 Extraction of thiaminase

Thiaminase extraction was performed with modifications of the method described by (Honeyfield et al., 2010a). Samples were homogenized with an approximately equal volume of dry ice by pulsing in a KRUPS coffee grinder (Model GX332) with a removable stainless-steel cup for 50 s and transferring to quart-sized plastic bags, which were then stored open at −20 °C overnight to allow the CO_2_ to sublimate. Samples were then transferred into 15 mL centrifuge tubes and stored at −80 °C until extraction. All sample homogenizations and tube transfers were performed in a 4 °C cold room. Four replicate samples of each tissue and processing step were homogenized, except for the pin bone sample from a single fish. We attempted a larger bone sample than from the pin bones, but processing it broke the shaft of the coffee grinder. TCEP buffer (100 mM potassium phosphate, 50 mM sodium chloride, 10 mM TCEP, corrected to pH 6.9 with sodium hydroxide and containing HALT™, a proprietary protease inhibitor (Thermo Fisher) at a 1:100 dilution) was freshly prepared each day using nitrogen sparged HPLC water. The TCEP buffer was sparged again on ice with N_2_ for at least 10 min before each extraction or assay. Approximately 1 g of the sample was weighed into 15 mL centrifuge tubes, to which was added ice-cold TCEP buffer at 5 mL per gram of sample. The samples were vortexed for 10 s at the highest speed three times, each time separated by 1-min rest on ice. Samples were then returned to the ice and allowed to settle for 10 min, though some samples required a centrifugation step (5 min at 2000 RCF) to settle the solids enough to retrieve a supernatant fraction. In particular, the outer bladder membrane samples that had been dehydrated and microwaved required twice the buffer volume and centrifugation to produce enough supernatant for further analysis. Sample concentration differences due to the excess buffer volume were later corrected for in downstream 4-NTP and Micro BCA™ assays. The supernatant was transferred to 1.5 mL centrifuge tubes and centrifuged for 20 min at 17,000 RCF, 4 °C. The resulting supernatants were transferred in 800 μL aliquots to 0.8 mL Pierce centrifuge columns containing ∼30 μm pore size polyethylene filters in new 1.5 mL centrifuge tubes and centrifuged a second time for 20 min at 17,000 RCF and 4 °C to remove any particulates. The filtrates were transferred to new 2.0 mL Protein Lo-Bind tubes (Eppendorf) and immediately assayed for thiaminase activity, with a portion of the filtrates set aside for Micro BCA™ protein quantification. The excess sample was stored at −80 °C. Our preliminary data indicated that thiaminase activity declined following extraction. Hence, as a precautionary measure, samples were assayed for thiaminase activity immediately after thiaminase extraction, and we included the HALT™ protease inhibitor in the extraction buffer.

### 2.4 Micro BCA protein quantification

Supernatant samples were analyzed for protein content using a Micro BCA™ Protein Assay Kit (Thermo Fisher). Briefly, samples were diluted 1:100 in phosphate-buffered saline (PBS) to bring their protein concentrations within the range of the Micro BCA™ assay (0.5–20 μg protein/mL), and 50 μL of the sample or supplied bovine serum albumin (BSA) standard was mixed with 100 μL of the BCA working reagent in each well of a clear Costar 3795 96-well plate. The plate was incubated at 37 °C for 2 h, then absorbance was read at 562 nm. The amount of protein was calculated based on the BSA standard curve.

### 2.5 Percent loss on drying

To determine the percent loss on drying, samples were weighed into weigh boats or microcentrifuge tubes, then stored in open containers in an oven at 55 °C for up to 48 h with weighing after 2, 4, 24, and 48 h. All samples were deemed dry by 48 h when the mass did not change by more than 0.05% from that collected at 24 h. The percent loss was calculated from the initial and final masses of material. This represents the total loss of mass upon drying and represents primarily moisture, but may potentially include other volatile substances.

### 2.6 4-NTP thiaminase assay

Thiaminase activity was measured with modifications of the method described in (Honeyfield et al., 2010a) on the same day as the thiaminase extraction. Freshly prepared and sparged TCEP buffer was added to the 96-well Costar 3795 plate wells at 24 μL per well, followed by 16 μL of the sample in triplicate. This additional buffer was included to aid sample recovery from the pipet tip upon its addition. 16 μL of sample at 1 g sample per 5000 μL volume yielded 0.0032 g sample per well. Positive controls consisted of 8 or 16 μL of 1 μg/mL recombinant thiaminase in TCEP buffer, and negative controls consisted of TCEP buffer alone. Reaction buffers were prepared by adding 4-NTP freshly reconstituted in DMSO at 200 mM to ice-cold TCEP buffer to yield an intermediate concentration of 279 μM. The 4-NTP TCEP buffer was immediately divided into two aliquots; one aliquot received 445 μM thiamine in TCEP buffer and the other an equivalent volume of TCEP buffer, both resulting in a final 4-NTP concentration of 267 μM. The buffer without added thiamine was used to obtain the baseline for correcting the thiamine-dependent rate of disappearance of 4-NTP. The reaction buffers were kept on ice and protected from light, and 100 μL was immediately added to each sample well. Absorbance at 411 nm was read at approximately 30-s intervals for 30 min in the Varioskan LUX Multimode Microplate Reader from Thermo Fisher Scientific that was pre-warmed and maintained at 37 °C. After 30 min, single absorbance measurements at 900 nm and 977 nm were determined for pathlength correction.

### 2.7 Calculation of enzyme activity

Pathlength correction was achieved by determining the pathlength of each well from the equation ((Abs_977_-Abs_900_)/0.173), where 0.173 equals the K-factor for weak aqueous buffers (Thermo Fisher Scientific, 2015). The Abs_411_ was then divided by the pathlength correction and plotted vs. elapsed time. For each sample time point, the baseline absorbance at t = 0 was subtracted. Each resulting time curve was then examined for aberrant behavior—if a value was positive at this step, indicating a gain in absorbance rather than the expected decline in signal, this curve was deemed invalid and was not analyzed further. The resulting value was converted to pmol 4-NTP using the extinction coefficient for 4-NTP in TCEP buffer (13,650 M^−1^cm^−1^) (Hanes et al., 2007) and correcting for sample dilution. Then the pmol amount from the sample containing 0 μM thiamine in the reaction buffer was subtracted from the pmol amount from its corresponding 445 μM thiamine sample and replicates were averaged. The slopes of these differences with respect to time were determined in 5-min increments. The maximal 5-min slope was determined in the range of 5–25 min. In many samples, there was significant variation during the first 5 min, likely due to thermal equilibration, so the data from zero to 5 min were not used. The slopes, in pmol/min. were converted to nmol/min. and expressed as specific activity using a ratio of 4-NTP concentration to either g sample (from weighing) or g protein (from the micro BCA™ assay) in the sample. When comparisons were made to positive or negative controls, the rate was maintained as pmol/min. since the controls did not have a corresponding gram of sample or protein weight.

### 2.8 Data handling

The biological replicates for both the protein concentration assays and the thiaminase activity assays were each analyzed in triplicate. Error bars in plots or ± values in the tables represent standard error of the mean as indicated in the figure legends. Determination of the slope values was carried out using GraphPad Prism, version 9.4.1 (GraphPad Software, San Diego, California USA, www.graphpad.com). For thiaminase measurements, rates that were below negative control were deemed nondetectable. Differences in protein concentration and thiaminase activity between raw tissue and viscera, skin, and bladder and separately to processed tissues were compared with significance determined using one-way ANOVA in GraphPad version 9.4.1. Results were deemed significant at a p ≤ 0.05.

## 3 Results and discussion

*Hypophthalmichthys molitrix* (silver carp) represents enormous biomass in the Mississippi River watershed, a threat to its ecosystem, and a continuing risk of invasion to other bodies of water (Garvey, 2015; Ivan et al., 2020). Thus, there is great interest in finding commercial uses for these fish to provide incentives for their removal from these bodies of water (Bowzer et al., 2013; Ferguson et al., 2020). One concern is thiamine-degrading activity due to the presence of the enzyme thiaminase, which has been previously noted in silver carp and other carp species (Arsan and Malyarevskaya, 1969; Bos and Kozik, 2000; Gnaedinger and Krzeczkowski, 1966; Greig and Gnaedinger, 1971; Krampitz and Woolley, 1944; Lee, 1948; Masako et al., 1994; Sealock et al., 1943; Spitzer et al., 1941). A solid understanding of the presence of thiaminase and viable techniques for reducing its possible activity is valuable for developing a market for this fish.

It was shown in early studies that foxes and chicks fed cooked fish did not suffer Chastek's syndrome or polyneuritis (Spitzer et al., 1941). A subsequent study indicated that heating for 5 min at 180 °F was the minimum condition to inactivate thiaminase in whole raw fish, but at least 7 min at the same temperature to inactivate thiaminase activity in viscera (Gnaedinger and Krzeczkowski, 1966). Thiaminase inactivation in fish tissues by the use of heat has been described in association with animal feeding studies (mink, foxes, and fish-eating birds), but these reports have not evaluated a variety of food processing methods (Heaton et al., 1995). To our knowledge, the effect of processing fish material by baking, microwaving, dehydrating, or freeze-drying on thiaminase activity has not been systematically investigated. The primary aims of this study were to determine the levels of thiaminase activity in *H. molitrix* tissues and to determine if routine processing steps successfully mitigate the potential activity. A previously developed colorimetric method was used to assess thiaminase activity, which relied on the depletion of the absorbance signal from 4-nitrothiophenol (4-NTP), an efficient thiaminase I cosubstrate, at 411 nm upon reaction with thiaminase I and thiamine (Hanes et al., 2007; Honeyfield et al., 2010a; Kraft et al., 2014).

### 3.1 Thiaminase activity per gram of sample in raw samples

Individual *H. molitrix* collected as part of the batches used in this study weighed, on average, approximately 10 pounds each and were two feet in length. Representative images of a single *H. molitrix* fish and raw *H. molitrix* samples that were used in the study are included in Fig. 1.

Raw tissue contained thiaminase activity ranging from undetectable in the bone, to 2.38 nmol/g sample/min. in the flesh, to 9.17 nmol/g sample/min. in the viscera (Table 1). These measured activities align with results observed in other fish species commonly associated with thiaminase activity (e.g.-alewife and rainbow smelt (*Osmerus mordax*), ranging from 1.8 to 39.8 nmol/g/min (Boggs et al., 2019; Tillitt et al., 2005; Wright et al., 2005)) and were significantly greater than activities observed in species not associated with thiaminase activity (e.g., yellow perch (*Perca flavescens*) and round goby (*Neogobius melanostomus*)*,* ranging from 6 to 26 pmol/g/min (Tillitt et al., 2005).) Thiaminase activity varies considerably within fish species temporally and spatially, which may reflect dietary composition, immune status, or other stressor conditions (Boggs et al., 2019; Honeyfield et al., 2012; Honeyfield et al., 2010b; Lepak et al., 2008; Lepak et al., 2013; Tillitt et al., 2005; Wistbacka and Bylund, 2008). Analytical methods for measurement of thiaminase activity and data analyses can also contribute to differences in reported values across publications (Hanes et al., 2007; Honeyfield et al., 2010b; Kraft et al., 2014; Zajicek et al., 2005).

**Table 1:** Thiaminase activity in raw samples of Silver carp (Hypophthalmichthys molitrix).

Tissue type	Thiaminase total activity (pmol/min.)	Thiaminase specific activity (nmol/g sample/min.)
Raw flesh	10.14 ± 8.24	3.17 ± 2.56
Raw flesh (second set of samples)	8.89 ± 5.27	2.78 ± 1.65
Viscera	29.34 ± 0.39	9.17 ± 0.12
Skin	7.62 ± 2.25	2.38 ± 0.70
Swim bladder	16.20 ± 1.72	5.06 ± 0.54
Bone	ND	ND

The g sample refers to the mass of sample ‘as is’, weighed without further drying or processing. All samples were shipped to the laboratory frozen, so they underwent one freeze-thaw cycle before analysis. Values in this table are presented as the mean with the standard error of four biological replicates. Rates that were less than that of the negative control were labeled as non-detectable (N.D.) Mean rates of thiaminase replicate assays are presented as total activity and specific activity on a per-gram-of-sample basis.

*Raw flesh –* Thiaminase activity was observed in raw flesh samples from *H. molitrix* (Table 1). These findings are in line with other reports of fish species commonly associated with thiaminase activity (Fujita 1954; Zajicek et al., 2005). Muscle tissues generally contain measurable amounts of thiaminase activity, but those activities are usually several fold less than internal organs and the gastrointestinal tract (viscera) (Arsan and Malyarevskaya, 1969; Gnaedinger and Krzeczkowski, 1966; Kraft et al., 2014; Spitzer et al., 1941; Woolley, 1941).

*Viscera* – Viscera contained the greatest specific activity of raw tissues (9.17 nmol/g sample/min.) from *H. molitrix* (Table 1). This was consistent with tissue distribution of thiaminase activity in other fish species, where activity was concentrated in the gastrointestinal tissues relative to other tissues or organs of the body (Arsan and Malyarevskaya, 1969; Gnaedinger and Krzeczkowski, 1966; Kraft et al., 2014; Spitzer et al., 1941; Woolley, 1941). This may be reflective of the composition of the microbiota or diet. Thiaminase activity in fish is often associated with microbial sources, specifically the gram-positive bacteria *Paenebacillus thiaminolyticus,* which has been found in the viscera of prey fish (Honeyfield et al., 2002; Richter et al., 2009; Zajicek et al., 2009). Silver carp are filter-feeders with a diet consisting mainly of phytoplankton (63.5%) and zooplankton (33.8%) (Tumolo and Flinn, 2019). Bacteria are known sources of thiaminases, and those fishes that are consumers of constituents lower in the food chain, including algae and cyanobacteria, have been associated with higher thiaminase activity (Arsan and Malyarevskaya, 1969; Boggs et al., 2019; Riley and Evans, 2008; Tumolo and Flinn, 2019). Early work suggested that increasing the composition of blue-green algae in the diet supplied to silver carp resulted in higher thiaminase activity measured in the liver and intestine (Arsan and Malyarevskaya, 1969). Alternatively, evidence suggests thiaminase can be produced *de novo* by certain species of fishes (Richter et al., 2023). If the function of the enzyme in fishes relates to immune status as suggested by Wistbacka and Bylund, (2008), elevated activity of thiaminase in the viscera of fish, as observed here, would be consistent with this notion.

*Skin, swim bladder, bones, and stickwater* – Skin from *H. molitrix* contained a significant amount of thiaminase activity, similar to the activity of the raw unprocessed flesh samples in this study (Table 1). Skin of common carp (*Cyprinus carpio*) was observed to have thiaminase activity similar to raw flesh and much less than internal organs or viscera (Fujita, 1954), consistent with our findings (Table 1). Thiaminase activity was also quite apparent in the swim bladder of Silver carp (Table 1). This finding is in contrast to an early study where no activity was observed in the swim bladder of Crucian carp (*Carassius carassius*) (Sealock et al., 1943). We assayed the exterior layer of the anterior portion (see Fig. 1), which may have affected our findings, as compared to using the entire organ. We hypothesize that the presence of activity may be microbial in origin and a function of the linkage of this organ to the digestive tract via the esophagus through the pneumatic duct. However, the presence of an endogenous thiaminase enzyme synthesized by the fish themselves rather than by dietary acquisition cannot be excluded (Richter et al., 2023). The sources of thiaminase activity in fish and, consequently, the nature of those enzymes remain to be more fully delineated. No thiaminase activity was observed in pin bones, the small bones radiating from the vertebrae found within the dorso-lateral musculature of silver carp. Stickwater, the water-soluble liquid rich in nutrients residual from fish processing and considered as a possible supplement to animal foods due to its nutritional content (Bechtel, 2005; Kousoulaki et al., 2009; Mahdabi and Hosseini Shekarabi, 2018), showed a high extracted protein content (8.38 ± 0.42 mg/mL), but no detectable thiaminase activity (Table 2).

**Table 2:** Thiaminase activity in processed samples from Silver carp (Hypophthalmichthys molitrix).

Processing step	Thiaminase total activity (pmol/min.)	Thiaminase specific activity (nmol/g sample/min.)
***Flesh***		
Raw flesh	10.14 ± 8.24	3.17 ± 2.56
Dehydrated, 160 °F, 5 h*	13.45 ± 2.13	4.20 ± 0.67
Dehydrated, 160 °F, 10 h*	11.17 ± 4.44	3.49 ± 1.39
Dehydrated, 160 °F, 20 h*	5.40 ± 1.58	1.69 ± 0.49
Dehydrated, 160 °F, 5 h	27.78 ± 4.84	8.68 ± 1.51
Dehydrated, 160 °F, 10 h	18.33 ± 2.07	5.73 ± 0.65
Baked 200 °F, 1 h	13.99 ± 2.36	4.37 ± 0.74
Baked 400 °F, 0.5 h	2.59 ± 1.32	0.81 ± 0.41
Baked 400 °F, 0.5 h*	ND	ND
Dehydrated, 160 °F, 20 h, Baked 200 °F 30 min.	10.69 ± 6.33	3.34 ± 1.98
Dehydrated, 160 °F, 20 h, Baked 300 °F 33 min.	5.66 ± 5.20	1.77 ± 1.62
Dehydrated, 160 °F, 20 h, Baked 400 °F 25 min.	ND	ND
Microwaved, 1.0 min.	3.62 ± 0.74	1.13 ± 0.23
Microwaved, 2.0 min.	2.80 ± 1.78	0.88 ± 0.56
Microwaved, 2.5 min.	2.70 ± 4.99	0.84 ± 1.56
Microwaved, 2.5 min*	5.06 ± 1.50	1.58 ± 0.47
Microwaved, 4.0 min.	ND	ND
Freeze-dried, 24 h	34.28 ± 4.13	10.71 ± 1.29
***Bladders***		
Inner bladder, dehydrated 160 °F, 20 h*	ND	ND
Outer bladder, dehydrated 160 °F, 20 h*	8.46 ± 3.46	2.64 ± 1.08
Outer bladder, Dehydrated 160 °F, 12 h	14.23 ± 1.48	4.45 ± 0.46
Outer bladder, Dehydrated 160 °F, 12 h Microwaved 3 min.	17.07 ± 2.31	5.34 ± 0.72
Outer bladder, Dehydrated 160 °F, 16 h Microwaved 3 min.	4.12 ± 2.13	1.29 ± 0.66
***Stickwater***		
Stickwater, frozen	ND	ND
Stickwater, Baked 400 °F, 15 min.	ND	ND
Stickwater, Baked 400 °F, 10 h	ND	ND
Stickwater, Baked 200 °F, 1 h	ND	ND
Stickwater, Baked 400 °F, 30 min*	3.56 ± 1.00	1.11 ± 0.31

The g sample refers to the mass of sample ‘as is’, weighed without further drying or processing. All samples were shipped to the laboratory frozen, so all underwent one freeze-thaw cycle before analysis. Those labeled with * were frozen as raw tissues prior to the listed processing steps and were frozen again prior to shipment. Values in this table are presented as the mean with the standard error of four biological replicates. Rates that were less than that of the negative control were labeled as non-detectable (N.D.) Mean rates of thiaminase replicate assays are presented as total activity and specific activity on a per-gram-of-sample basis.

It is important to note that thiaminase is suspected or present in several fish species, including anchovy and smelt (Greig and Gnaedinger, 1971; NOAA Fisheries, 2021; Neilands, 1947; Stout et al., 1963), that are also consumed raw, dehydrated, or freeze-dried by people and offered as food to domesticated pets. We postulate that the source of thiaminase (microbial or *de novo* in the fish, which is beyond the scope of our current work) may be an important factor in its ability to produce thiamine deficiency. The low pH environment and proteolytic enzymes of the stomach of many carnivorous mammals (Beasley et al., 2015), including humans, would be expected to inactivate thiaminase released as free protein. By contrast, microbially produced thiaminases may be afforded some protection through entrapment during passage, and the microbial producers could potentially reach and colonize the intestine. This would seem likely in higher gastric pH environments where proteolytic enzymes are less efficient and bacterial colonization is more likely (Tennant et al., 2008). Other factors, including the thiamine status of the host and of the diet itself, including initial thiamine content, percentage of thiaminase-containing fish, availability of thiaminase I cofactors or inhibitors, and pH are likely to play a role in susceptibility to thiaminase-mediated thiamine depletion (Edwards et al., Unpublished results; Fitzsimons et al., 2005; Zajicek et al., 2009).

### 3.2 Thiaminase activity per gram of sample in processed samples

**Fig. 2:**
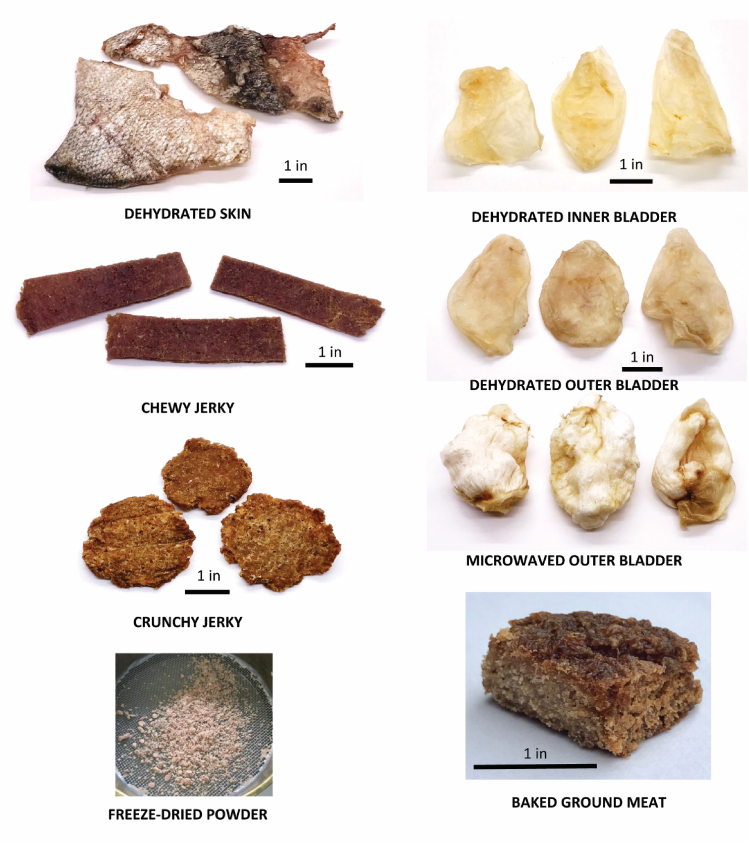
Representative images of select processed samples which include dehydrated skin, chewy jerky, crunchy jerky, freeze dried powder, dehydrated inner bladder, dehydrated outer bladder, outer bladders after microwaving, and baked ground flesh.

Representative images of processed *H. molitrix* samples that were used in the study are included in Fig. 2.

**Fig. 3:**
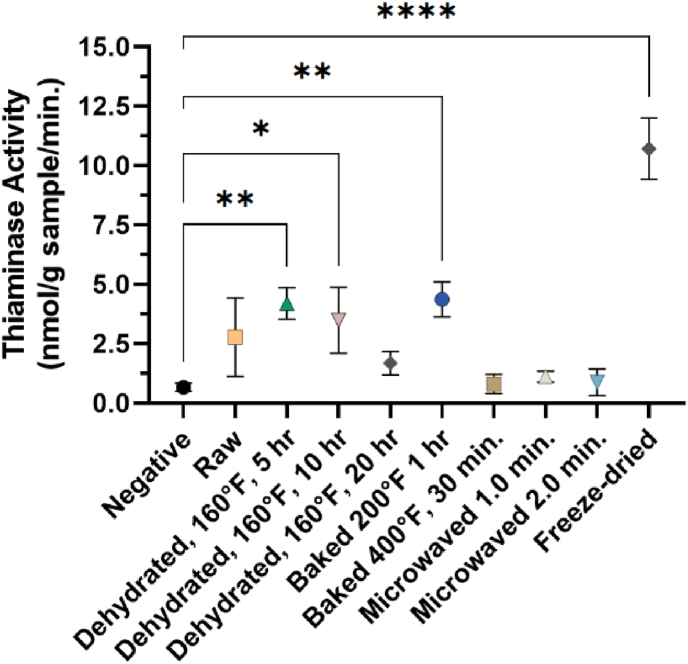
Thiaminase activity (nmol/g sample/min.) in raw and processed flesh samples, normalized on a per-gram-of-sample basis. The results are the average of four biological replicates with error bars representing their standard error. Significance was tested relative to the apparent rate of degradation from the negative control using a one-way ANOVA. *p ≤ 0.05, **p ≤ 0.01, and ****p ≤ 0.0001.

Samples of ground flesh that had been microwaved at 950 W for one or more minutes showed reductions in thiaminase activity versus raw tissues comparable to the negative control (Fig. 3, Table 2). By contrast, baking the tissues at 200 °F for 1 h or dehydrating the samples for five or 10 h at 160 °F appeared to have a slight concentration effect, causing an increase in thiaminase activity. These forms of processing resulted in water loss upon drying (Table 3 and S1), but the temperatures used were inefficient at inactivating the enzyme. However, extending the dehydration to 20 h reduced the activity significantly, and baking at 400 °F for 30 min was sufficient to reduce the activity to undetectable levels. The activity in dehydrated samples could be reversed and negated by baking at 400 °F. Of the processing conditions used, microwaving and prolonged dehydration (16 h at 160 °F) were required to reduce the activity in the outer bladder to undetectable levels (Table 2).

Most notably, freeze-drying significantly increased thiaminase activity by approximately fourfold over that of raw tissues. This form of processing would not subject the enzyme to elevated temperatures and would be unlikely to inactivate it but rather act to preserve its activity.

The results with various processing steps on the thiaminase activity (Fig. 3) indicated that thiaminase activity can be easily negated in *H. molitrix* in final processed products by microwaving or baking under certain conditions. However, the time and temperature dependence must be considered to avoid concentrating, but not inactivating, the enzyme.

### 3.3 Protein extraction efficiency and degree of hydration

Our initial inquiry into thiaminase activity focused on the rate of thiamine degradation as a function of sample mass, which has been the standard practice in thiaminase activity assays in raw tissues (Honeyfield et al., 2012; Kraft et al., 2014; Tillitt et al., 2005; Wright et al., 2005; Zajicek et al., 2005). The mass of material is a logical comparator across different tissue types and sources and would also be the primary measure of the amount for animal and human consumption. However, as this assay assesses enzyme activity in extracts of the products, and these extracts can vary significantly in terms of their degree of hydration and consistency, we deemed it of methodological interest to also consider the protein extraction efficiency.

To examine the degree of hydration within, the raw and processed tissue samples were weighed, dried for up to 48 h at 55 °C, then reweighed until a constant mass was obtained to determine the percent loss on drying (Table 3). The extracts used directly in the 4-NTP assay were measured using a bicinchoninic acid (BCA) assay to assess their protein content (Table 3). Results from additional processing variations are available in Tables S1 and S2, respectively.

**Table 3:** Percent loss of mass on drying and extracted protein concentration.

Processing step	% Loss upon drying	Protein Concentration (mg/mL)
Raw	76.2 ± 0.1	5.109 ± 0.490
Viscera	70.5 ± 0.4	2.765 ± 0.144
Skin	49.7 ± 0.5	1.125 ± 0.107
Outer bladder	39.0 ± 0.7	0.903 ± 0.268
Stick Water	92.3 ± 0.3	8.379 ± 0.417
Dehydrated, 160 °F, 10 h	3.5 ± 1.2	7.255 ± 0.359
Microwaved, 950 W, 4 min.	52.2 ± 1.2	1.500 ± 0.072
Freeze-dried, 24 h	0.6 ± 0.6	23.330 ± 0.375
Baked, 200 °F, 1 h	73.2 ± 1.8	1.245 ± 0.058
Baked, 400 °F, 0.5 h	68.1 ± 0.3	1.282 ± 0.046

The mean value of triplicate samples is presented with ±values representing the standard error.

**Fig. 4:**
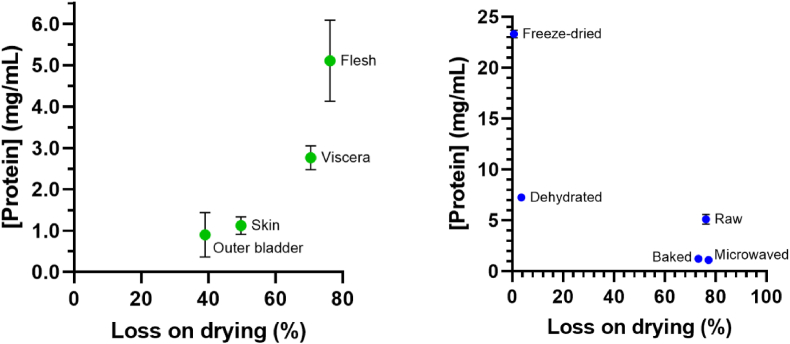
Relationship between percent loss of mass upon drying and the concentration of extractable protein per tissue type as determined using the microBCA assay. A.) Samples were the outer bladder, skin, viscera, and flesh. B.) Samples of flesh were raw, microwaved for 2.5 min at 1 kW, baked at 200 °F for 1 h, dehydrated at 160 °F for 10 h, or freeze-dried for 24 h. Each bar represents the average of four replicates, with the error bars representing their standard error.

With raw tissues, we observed significant variation tissue to tissue on the mass loss on drying (Fig. 4a). We observed a 76.2% loss upon drying (23.8% mass remaining) for raw flesh yielding a 5.1 mg/mL extracted protein concentration versus 39.0% loss on drying (61.0% mass remaining) for the outer bladder corresponding to a 0.9 mg/mL extracted protein concentration. These results indicated that although a tissue contained a higher percent wet weight, indicating less solid tissue mass and possibly more significant dilution of the extracted protein, this did not necessarily correspond to lower protein concentrations and vice versa. Our loss on drying results for raw flesh (76.2%) are consistent with those carried out by a commercial laboratory on a separate batch (73.1%) (Table S3).

For processed tissue, we found that the amount of protein released under identical extraction conditions from baked, microwaved, dehydrated, or freeze-dried samples differed significantly from each other and raw, unprocessed tissue (Fig. 4b). We found that freeze-dried tissue yielded minimal loss on drying (0.6%) and a high extracted protein concentration (23.3 mg/mL) compared to raw tissue. Our results, indicating a 4.7-fold difference in extractable protein in raw tissue versus freeze-dried tissue, were consistent with the 3.7-fold difference from an independent nutritional laboratory found in a separate batch of *H. molitrix* (reported 20.2% protein in raw ground tissue versus 74.8% protein in freeze-dried powder) (Table S3.) By contrast, microwaving or baking the tissue yielded a significant loss on drying (52–73%) and a low extractable protein concentration ranging from 1.0 to 1.5 mg/mL.

These results indicated that processing steps, including microwaving or baking, retained a significant amount of the tissue water but reduced the amount of releasable protein. In contrast, freeze-drying significantly reduced the amount of tissue water, yielding little further water loss upon drying but a marked increase in extracted protein concentration. In dehydrated samples containing similarly low residual moisture to freeze-drying, only one-third of the protein was obtained in the extracts (7.3 mg/mL) versus that from freeze-dried samples.

The collective results indicate that while water loss was a factor in protein extraction efficiency, the tissue type and the consistency of the tissue following processing can result in significantly different protein concentrations under the same extraction conditions. The change in protein extraction efficiency would directly impact the evaluation of the measured activity of thiaminase I, wherein a low protein extraction efficiency would inherently yield low observed thiaminase activity in a sample weighed at the same wet weight basis and vice versa.

Varied processing steps were shown to have different levels of hydration, which can potentially concentrate the protein while simultaneously making the release of the protein from the sample matrix more difficult. This was reflected in our loss upon dehydration and protein concentration data, where differences of up to 77-fold and 20-fold were observed, respectively (Fig. 4, Table 3, and Tables S1 and S2).

### 3.4 Thiaminase activity per gram of protein

When our thiaminase activity data were instead normalized on a per-gram-of-protein basis, reflecting the efficiency of protein extraction, we observed the most significant difference between raw tissues and processed tissues with those that were baked at 200 °F for 1 h (Tables S4 and S5). This processing condition was ineffective at reducing thiaminase activity, consistent with the results on a per-gram-of-sample basis. The results on a per-gram-of-protein basis indicate that baking samples at 200 °F for 1 h significantly reduced the amount of extractable protein. However, what was released, including thiaminase, remained enzymatically active. By contrast, increasing the baking temperature to 400 °F for 30 min resulted in similar extracted protein concentrations but low thiaminase activity, indicating the effectiveness of this condition in inhibiting thiamine degradation activity. The freeze-dried samples on a per-gram-of-sample basis had very high activity relative to raw samples, but these samples were not significantly different on a per-gram-of-protein basis. The protein-normalized thiaminase activity in raw samples was not significantly different from freeze-dried samples since the extractable protein content in the latter was also much higher. This normalization indicated that the thiaminase did not lose activity during freeze-drying.

The viscera retained a high activity level in raw samples, irrespective of the normalization approach. The high activity levels in the viscera are consistent with the protein-normalized results in alewife viscera previously reported (Gnaedinger and Krzeczkowski, 1966). Protein-normalized activity levels in the skin and outer bladder were more pronounced than at activity normalized by gram of sample. This was reflective of their relatively high activity but low extracted protein concentrations.

## 4 Conclusions

Here we confirm that the invasive silver carp (*Hypophthalmichthys molitrix*) contains measurable levels of thiaminase activity in most tissues studied and present data that may guide processes to destroy thiaminase activity before its commercial use in food for humans or pets. Our results suggest that baking at elevated temperatures or microwaving the tissue is warranted in products where thiaminase activity needs to be significantly reduced or at undetectable levels. Dehydration of products yielded an apparent concentration effect on the enzyme without its inactivation, requiring extended dehydration processes to reduce the activity. Freeze-drying was ineffective at reducing activity and appeared to instead concentrate and preserve the enzyme. In this work, we also extended the use of the 4-NTP method for assessing thiaminase activity in processed tissues. For such application, we suggest that the degree of hydration and protein extraction efficiency are also considered, rather than only sample mass, as the measured activity would vary significantly in raw versus processed tissues. Freeze-dried and dehydrated samples showed significantly higher concentrations of total extracted protein in the supernatants, paralleling the greater amount of fish material present in a constant mass of dried fish versus raw fish or either microwaved or baked samples. This speaks to the likely increased availability of proteins to the consumer for fish processed by these methods. However, it also suggests caution in supplying *H. molitrix* and potentially other thiaminase-producing fish processed by these methods as a significant dietary source without a further understanding of thiamine depletion. Our results focus on thiaminase I activity levels and do not include measures of thiamine content following these various processes, which is a subject warranting further work given the known instability of thiamine in food processing.

## Disclaimer

Any use of trade, firm, or product names is for descriptive purposes only and does not imply endorsement by the U.S. Government.

## CRediT authorship contribution statement

**Patricia C. Wolfe:** Formal analysis, Writing – original draft. **Amber M. Tuske:** Investigation, Writing – review & editing. **Donald E. Tillitt:** Writing – original draft. **Fred Allen:** Writing – original draft, Resources. **Katie A. Edwards:** Supervision, Resources, Formal analysis, Writing – original draft.

## Declaration of competing interest

The authors declare the following financial interests/personal relationships which may be considered as potential competing interests: PW, DT, AT and KE declare no competing interests. FA is a consultant for Two Rivers Fisheries (TRF) and personally provided partial financial support to the laboratory of KE for supplies. Neither TRF nor FA was involved in data analyses, interpretation or presentation.

## Data Availability

Data will be made available on request.
