# Supplementary material for: Understanding and mitigating thiaminase activity in silver carp
Source: Curr Res Food Sci. 2023 Apr 7;6:100502. doi: 10.1016/j.crfs.2023.100502 (PMC10290998; doi:10.1016/j.crfs.2023.100502)
Supplement: Multimedia component 1 [file mmc1.docx]

**Understanding and Mitigating Thiaminase Activity in Silver Carp**

Patricia C. Wolfe^1^, Amber M. Tuske^1^, Donald E. Tillitt^2^, Fred Allen^3^, Katie A. Edwards*^,1^

^1^Department of Pharmaceutical Sciences, Binghamton University, Binghamton, NY, 13902

^2^ U.S. Geological Survey, Columbia Environmental Research Center, Columbia, MO 65201

^3^RADii Solutions, LLC, Princeton, NJ 08540 / Carpe Eat’m, LLC, Paducah, KY 42001

Table S1. Percent loss of mass on drying

| **Processing step** | **% Loss on drying** |
| --- | --- |
| Raw | 76.2±0.1 |
| Viscera | 70.5±0.4 |
| Skin | 49.7±0.5 |
| Outer bladder | 39.0±0.7 |
| Stickwater | 92.3±0.3 |
| Dehydrated, 160˚F, 10 hr | 3.5±1.2 |
| Baked 200˚F, 1 hr | 73.2±1.8 |
| Baked 400˚F, 0.5 hr | 68.1±0.3 |
| Baked 400˚F, 0.5 hr* | 57.7±0.7 |
| Dehydrated, 160˚F, 20 hr, Baked 300˚F 33 min. | 1.5±0.9 |
| Microwaved, 1.0 min.** | 68.8 |
| Microwaved, 2.5 min.* | 77.2±2.7 |
| Microwaved, 4.0 min. | 52.2±1.2 |
| Freeze-dried, 24 hr* | 0.6±0.6 |
| Outer bladder, Dehydrated 160˚F, 20 hr | 7.7±0.9 |
| Outer bladder, Dehydrated 160˚F, 12 hr | 2.6±0.8 |
| Outer bladder, Dehydrated 160˚F, 12 hr Microwaved 3 min. | 2.2±0.9 |

*All samples were shipped to the laboratory frozen, so all underwent one freeze-thaw cycle before analysis. Those labeled with * were frozen as raw tissues prior to the listed processing steps and were frozen again prior to shipment. The mean value of triplicate samples is presented with ± values representing the standard error. **This sample was a single point determination for loss on drying.* Stickwater is the liquid that drains from the raw or frozen ground fish upon thawing and is sometimes used in commercial animal meals.

Table S2. Extracted protein concentration

| **Processing step** | **Protein conc. (mg/mL)** |
| --- | --- |
| Raw | 4.973±0.220 |
| Raw | 5.109±0.490 |
| Viscera | 2.765±0.144 |
| Skin | 1.125±0.107 |
| Outer bladder | 0.903±0.268 |
| Dehydrated, 160˚F, 5 hr* | 6.061±0.412 |
| Dehydrated, 160˚F, 10 hr* | 5.003±0.153 |
| Dehydrated, 160˚F, 20 hr* | 4.341±0.430 |
| Dehydrated, 160˚F, 5 hr | 8.791±0.446 |
| Dehydrated, 160˚F, 10 hr | 7.255±0.359 |
| Baked 200˚F, 1 hr | 1.245±0.058 |
| Baked 400˚F, 0.5 hr | 1.282±0.046 |
| Baked 400˚F, 0.5 hr* | 1.597±0.051 |
| Dehydrated 20 hr, Baked 200˚F 30 min. | 3.989±0.221 |
| Dehydrated 20 hr, Baked 300˚F 33 min. | 2.492±0.145 |
| Dehydrated 20 hr, Baked 400˚F 25 min. | 2.028±0.075 |
| Microwaved, 1.0 min. | 1.159±0.161 |
| Microwaved, 2.0 min. | 1.487±0.042 |
| Microwaved, 2.5 min. | 1.107±0.188 |
| Microwaved, 2.5 min.* | 1.163±0.101 |
| Microwaved, 4.0 min. | 1.500±0.072 |
| Freeze-dried, 24 hr | 23.330±0.375 |
| Inner bladder, Dehydrated 160˚F 20 min.* | 3.430±0.410 |
| Outer bladder, Dehydrated 160˚F 20 min.* | 6.424±0.226 |
| Outer bladder, Dehydrated 160˚F, 12 hr | 2.418±0.128 |
| Outer bladder, Dehydrated 160˚F, 12 hr Microwaved 3 min. | 4.459±0.256 |
| Outer bladder, Dehydrated 160˚F, 16 hr Microwaved 3 min. | 1.894±0.072 |
| Stickwater, Frozen | 8.379±0.417 |
| Stickwater, Baked 400˚F, 15 min. | 1.541±0.026 |
| Stickwater, Baked 400˚F, 10 hr | 1.927±0.150 |
| Stickwater, Baked 200˚F, 1 hr | 1.671±0.266 |
| Stickwater, Baked 400˚F, 30 min. | 1.758±0.196 |

*All samples were shipped to the laboratory frozen, so all underwent one freeze-thaw cycle before analysis. Those labeled with * were frozen as raw tissues prior to the listed processing steps and were frozen again prior to shipment. The mean value of samples analyzed in quadruplicate is presented with ± values representing the standard error.*

Table S3. Nutritional analysis of Silver carp (*Hypophthalmichthys molitrix*) raw ground meat versus freeze-dried powder

| **Nutritional Analysis*** | **Raw Ground Meat** | **Freeze-Dried Powder** | **Method** |
| --- | --- | --- | --- |
|  | **Concentration wt%** | |  |
| Moisture (vacuum oven) | 73.1 | 2.2 | AOAC 950.46 (mod) |
| Protein | 20.2 | 74.8 | MWL FO 014 |

* Analytical data obtained by NutriData, Laguna Hills, CA and Midwest Laboratories, Omaha, NE

***Table S4. Thiaminase activity in raw samples from Silver carp (*Hypophthalmichthys molitrix)**

| **Tissue type** | **Thiaminase total activity (pmol/min.)** | **Thiaminase specific activity (nmol/g protein/min.)** |
| --- | --- | --- |
| Raw flesh | 10.14±8.24 | 127.39±103.68 |
| Raw flesh (second set of samples) | 8.89±5.27 | 108.74±65.32 |
| Viscera | 29.34±0.39 | 664.42±35.59 |
| Skin | 7.62±2.25 | 423.12±131.09 |
| Swim bladder | 16.20±1.72 | 1120.81±353.72 |
| Bone | ND | ND |

*All samples were shipped to the laboratory frozen, so they all underwent one freeze-thaw cycle before analysis. Those labeled with * were frozen as raw tissues prior to the listed processing steps and were frozen again prior to shipment. Values in this table are presented as the mean with the standard error of four biological replicates. Rates that were less than that of the negative control were labeled as non-detectable (ND) Mean rates of thiaminase replicate assays are presented as total activity and specific activity on a per-gram-of-protein basis.*

***Table S5. Thiaminase activity in processed samples from Silver carp* Hypophthalmichthys molitrix)**

| **Processing step** | **Thiaminase total activity (pmol/min.)** | **Thiaminase specific activity (nmol/g protein/min.)** |
| --- | --- | --- |
| *Flesh* | | |
| Raw flesh | 10.14±8.24 | 127.39±103.68 |
| Dehydrated, 160˚F, 5 hr* | 13.45±2.13 | 138.71±23.93 |
| Dehydrated, 160˚F, 10 hr* | 11.17±4.44 | 139.57±55.66 |
| Dehydrated, 160˚F, 20 hr* | 5.40±1.58 | 77.70±23.98 |
| Dehydrated, 160˚F, 5 hr | 27.78±4.84 | 197.46±35.83 |
| Dehydrated, 160˚F, 10 hr | 18.33±2.07 | 157.96±19.46 |
| Baked 200˚F, 1 hr | 13.99±2.36 | 588.36±100.74 |
| Baked 400˚F, 0.5 hr | 2.59±1.32 | 107.72±55.09 |
| Baked 400˚F, 0.5 hr* | ND | ND |
| Dehydrated, 160˚F, 20 hr, Baked 200˚F 30 min. | 10.69±6.33 | 167.46±99.61 |
| Dehydrated, 160˚F, 20 hr, Baked 300˚F 33 min. | 5.66±5.20 | 142.01±130.63 |
| Dehydrated, 160˚F, 20 hr, Baked 400˚F 25 min. | ND | ND |
| Microwaved, 1.0 min. | 3.62±0.74 | 195.28±48.20 |
| Microwaved, 2.0 min. | 2.80±1.78 | 117.82±74.81 |
| Microwaved, 2.5 min. | 2.70±4.99 | 145.56±269.94 |
| Microwaved, 2.5 min.* | 5.06±1.50 | 271.82±84.09 |
| Microwaved, 4.0 min. | ND | ND |
| Freeze-dried, 24 hrs | 34.28±4.13 | 91.84±11.15 |
| *Bladders* | | |
| Inner bladder, dehydrated 160˚F, 20 hr* | ND | ND |
| Outer bladder, dehydrated 160˚F, 20 hr* | 8.46±3.46 | 82.32±33.75 |
| Outer bladder, Dehydrated 160˚F, 12 hr | 14.23±1.48 | 367.90±43.06 |
| Outer bladder, Dehydrated 160˚F, 12 hr Microwaved 3 min. | 17.07±2.31 | 239.38±35.23 |
| Outer bladder, Dehydrated 160˚F, 16 hr Microwaved 3 min. | 4.12±2.13 | 135.81±70.36 |
| *Stickwater* | | |
| Stickwater, frozen | ND | ND |
| Stickwater, Baked 400˚F, 15 min. | ND | ND |
| Stickwater, Baked 400˚F, 10 hr | ND | ND |
| Stickwater, Baked 200˚F, 1 hr | ND | ND |
| Stickwater, Baked 400˚F, 30 min.* | 3.56±1.00 | 126.61±38.12 |

*All samples were shipped to the laboratory frozen, so they all underwent one freeze-thaw cycle before analysis. Those labeled with * were frozen as raw tissues prior to the listed processing steps and were frozen again prior to shipment. Values in this table are presented as the mean with the standard error of four biological replicates. Rates that were less than that of the negative control were labeled as non-detectable (ND) Mean rates of thiaminase replicate assays are presented as total activity and specific activity on a per-gram-of-protein basis.*

**Disclaimer:** Any use of trade, firm, or product names is for descriptive purposes only and does not imply endorsement by the U.S. Government.
